# The preliminary development and psychometric properties of the Psychotherapy Side Effects Scale

**DOI:** 10.1002/brb3.2885

**Published:** 2023-01-09

**Authors:** Chen Fazhan, Liu Liang, Zhao Xudong, Feng Qiang, Ge Congcong, Zhao Yunhan

**Affiliations:** ^1^ Clinical Research Center for Mental Disorders, Chinese‐German Institute of Mental Health, Shanghai Pudong New Area Mental Health Center, School of Medicine Tongji University Shanghai P.R. China; ^2^ Department of Clinical Psychology, Shanghai East Hospital, School of Medicine Tongji University Shanghai P.R. China

**Keywords:** psychometric properties, psychotherapy, psychotherapy side effects scale, side effects

## Abstract

**Background:**

Side effects in psychotherapy are common and have a negative impact on patients or clients. However, effective evaluation tools are still lacking and have not been fully studied. The present study aims to develop a scale with good reliability and validity to measure the side effects of psychotherapy.

**Methods:**

The 25 items in the Psychotherapy Side Effects Scale (PSES) were condensed and distributed to 420 subjects online to test its psychometric properties.

**Results:**

The internal consistency of the PSES was satisfactory to excellent (Cronbach's *ɑ* coefficient was .95, and the Guttman split‐half coefficient was 0.88). A statistically significant negative correlation between the satisfaction score and the total score of the PSES was shown (*r* = −0.51, *p* < .001). The PSES could effectively discriminate between two groups with and without side effects (*F* = 250.95, *p* < .001) and was able to predict the occurrence of side effects in psychotherapy with an area under curve of 0.932 and a 95% confidence interval of 0.900–0.964 (*p* < .001). A cutoff was set at 36 points in total PSES score, from which the maximum Youden's index (= 0.72) could be obtained. The positive rate of the PSES was 24% (101/420).

**Conclusion:**

The PSES showed good internal consistency, content validity, concurrent validity, discriminant validity and predictive validity in evaluating and identifying side effects in psychotherapy. More advanced reliability testing methods and structural validity testing for PESE need to be practiced in the future to better serve clinical practice.

## 1 INTRODUCTION

Psychotherapy is an effective treatment for mental disorders (Dragioti et al., 2017). Like other medical treatments, psychotherapy may also have side effects that are inconsistent with the goals of treatment or may even cause harm (Linden & Schermuly‐Haupt, 2014). As early as the 1960s, some scholars reported the antitherapeutic side effects (Talbot et al., 1964) and deterioration effect (Bergin, 1966) of psychotherapy. Unfortunately, after more than half a century, the side effects of psychotherapy have not received enough attention. A review showed that only 21% of psychological interventions indicated the information on harm for patients, and only 4% of the trials reported adverse events (Jonsson et al., 2014). Only 10%–28% of therapists were aware of side effects in psychotherapy (Boisvert & Faust, 2003, 2006), and nearly 80% of the negative outcomes of psychotherapy were not identified effectively (Hatfield et al., 2010). More importantly, there is almost no professional information concerning the side effects of psychotherapy in the process of training therapists (Alexander et al., 2018). Meanwhile, an increasing number of studies have shown that the side effects of psychotherapy are common and have a negative impact on patients or clients (Crawford et al., 2016; Gerke et al., 2020; Yao et al., 2020). There is also increasing interest in determining specific negative effects of a given psychotherapy. For instance, patients with chronic depression and treatment resistance who received Cognitive Behavioral Analysis System of Psychotherapy (CBASP) reported negative effects of up to 92.3% and dependence on their therapist of 45.2% (Herzog et al., 2021). The lack of standardized definitions and effective evaluation tools may be the main reasons that the side effects of psychotherapy have not been fully studied (Herzog et al., 2019).

The definition of side effects in psychotherapy was first described clearly by Linden and Schermuly‐Haupt (2014). First, the side effects must be “unwanted events” inconsistent with the goal of psychotherapy that occur in parallel with or in the course of psychotherapy, and cause negative effects on the patients. Second, side effects are caused by ethical psychotherapy. Moreover, although the side effects have pressure or burden on patients, they do not necessarily hinder the achievement of treatment goals, even sometimes help to achieve the goals. Linden and colleagues made excellent attempts to develop psychotherapy side effect assessments, but most of their results and assessment tools were written in German and have not been popularized and applied (Linden et al., 2015). The Unwanted Effects–Adverse Treatment Reaction checklist (UE‐ATR) was introduced by Linden, but its psychometric properties were not reported (Linden, 2013).

Other instruments have been developed to assess the potential side effects of psychotherapy. For example, the Inventory for the Assessment of Negative Effects of Psychotherapy (INEP, Ladwig et al., 2014), the Positive and Negative Effects of Psychotherapy Scale (PANEPS, Steffen et al., 2019), and the Negative Effects Questionnaire (NEQ, Rozental et al., 2019) focused on the negative effects experienced by patients. Nevertheless, their items were generated from the literature review, the expert consensus, and the qualitative data taken from specific individuals with depressive and anxiety disorders. More importantly, negative effects of psychotherapy include the harm by the malpractice or unethical behaviors, which are not side effects. The Side Effects of Psychotherapy Scale (SEPS, Moritz et al., 2015) was also only tested in patients with obsessive‐compulsive disorder. Thus, wide application may be limited by the nature of the study samples. Recently, the Edinburgh adverse effects of psychological therapy (EDAPT, Glanaghy et al., 2021) scale was established based on a qualitative study, but the psychometric properties have not been reported. As with assessment of the effectiveness of psychotherapy, use of standardized instruments may help to decrease potential under reporting and allow for a more accurate assessment of potential harm. Regrettably, the existing tools for assessing the side effects of psychotherapy have not reached a consensus on the most important domains, and the psychometric properties of these instruments were not good (Herzog et al., 2019).

In addition, the accurate measurement to psychotherapy side effects can reduce the therapist bias effect (Waller & Turner, 2016), which may lead the therapist to abandon effective treatments that are considered to be harmful for patients. Identifying and managing the side effects in psychotherapy is a valuable indicator for an ethical therapist, which can significantly improve the effectiveness of treatment (Parry et al., 2016; Scott & Young, 2016; Whipple et al., 2003). In conclusion, it is necessary to develop a set of high‐quality psychological scales to measure side effects in psychotherapy.

Our team took the lead in carrying out qualitative research on the side effects of psychotherapy in China. In our previous research results (Qiang et al., 2020), four kinds of first‐order themes and 14 kinds of second‐order themes applying to psychotherapy side effects were extracted, which provided a good basis for developing a new scale for evaluating side effects. This study posed the following research hypotheses: (1) some experiences reported by patients or clients could be used to evaluate the side effects of psychotherapy; (2) these items taken from the experiences mentioned above could be integrated to form a scale with good reliability and validity to measure the side effects of psychotherapy; and (3) the higher the satisfaction of psychotherapy, the lighter the side effects experience should be.

## 2 MATERIALS AND METHODS

### 2.1 Scale development

In our previous qualitative research (Qiang et al., 2020), the themes applying to psychotherapy side effects were extracted clearly (Table 1). These 14 types of side effects belong to four dimensions of “pressure from the therapeutic relationship,” “emergence of new symptoms,” “problem unsolved,” and “pressure outside the treatment context.” Among them, the “special affection in the therapeutic relationship” refers to the love of the client to the therapist, and the “maladaptive behavior” refers to these behaviors that have a negative impact on oneself or others, such as overeating and self harm. In addition, the “symptom persistence” refers to those symptoms persist in psychotherapy and are not reduced or alleviated.

**TABLE 1:** Themes applying to side effects in psychotherapy (Qiang et al., 2020)

First‐order themes		Second‐order themes	
Code	Themes	Code	Themes
1.1	Pressure from the therapeutic relationship	2.1.1 2.1.2 2.1.3 2.1.4	Therapeutic relationship dependence Therapeutic relationship intensity Special affection in the therapeutic relationship Therapeutic relationship rupture
1.2	Emergence of new symptoms	2.2.1 2.2.2 2.2.3 2.2.4 2.2.5	Suicidal thoughts or behaviors Negative emotions Bad behavior Psychosomatic symptoms Stigma
1.3	Problem unsolved	2.3.1 2.3.2 2.3.3	Symptom persistence Symptom deterioration Harm from unresolved symptoms
1.4	Pressure outside the treatment context	2.4.1 2.4.2	Family tension Impaired social relationships

Based on these themes, the Psychotherapy Side Effects Scale (PSES) was preliminarily developed. First, an item pool was built according to the second‐order themes, with each secondary theme being expanded into 1–3 questions to assess the patient's experience. Twenty‐eight entries were carefully generated using a description regarding psychometric habits and cultural background. The developers were major in the theoretical orientation of systemic family therapy. Second, three experts in the field of psychotherapy were invited to review the content of the item pool to improve content validity. Three entries were combined according to expert recommendations. The theoretical schools of the three experts were psychodynamics, cognitive behavioral therapy, and systemic family therapy. Finally, the first version of the 25‐item PSES was tested in application to 10 patients to modify the expressions used in the sentences.

Subsequently, 25 items were generated, characterized by therapeutic relationship pressure, deteriorating or unsolved issues, pressure on family and social relations, novel symptoms including depression, anxiety, psychotic symptoms, or symptoms of hypomania, and ideas and behaviors inclining toward suicide or self‐harm, and stigma. The PSES was designed as a self‐report scale. At the beginning of the PSES, the following question was asked: “Is the psychotherapy you are currently undergoing causing you to have the following experience?”. Subjects rated the negative effects of each item according to their own experience as “not at all,” “mild,” “moderate,” “severe,” and “extremely severe.” Participants were asked to respond on a five‐point Likert scale (1 = not at all, 2 = mild 3 = moderate, 4 = severe, 5 = extremely severe), and the instrument contained no reverse‐scored items. The sum of the scores of each item constituted the total score of the PSES, which was used to evaluate the overall severity of side effects.

### 2.2 Side effects experience

Meanwhile, the following question was asked separately after the PSES to determine whether patients experienced side effects: “Through the overall presentation of these items, do you think that psychotherapy has caused side effects for you?”. “Yes” meant that there were side effects experience, and “no” meant that there were no side effects experience. To date, there is no standardized tool for the diagnosis of side effects in psychotherapy. The measurement was used to test the predictive validity of the scale. In other words, predictive validity measures the ability of the PSES to predict the real side effects experience of the subject.

### 2.3 Satisfaction evaluation

Because no accepted scales of side effects in psychotherapy have been developed, patient satisfaction with psychotherapy was measured to test the concurrent validity of the PSES. Participants rated satisfaction on a scale of 1–10 according to their own experience in psychotherapy in response to the following question: “What is your satisfaction with the psychotherapy you are currently receiving?”. The higher the score was, the higher satisfaction was. If satisfaction was rated as 1, it meant that the patient was very dissatisfied, and if satisfaction was rated as 10, it meant that the patient was very satisfied.

### 2.4 Data collection

The instrument was edited via the internet using an interface for administering surveys (Wen Juan Xing; www.wjx.cn). Demographic and psychotherapy characteristics for the sample were also released alongside the PSES. The detailed information collected is shown in Table 3.

The edited online investigation was released on the WeChat platform on February 7, 2021. Participants read and decided whether to complete the questionnaire according to the inclusion criteria and gave informed consent before submitting the questionnaire. The investigation was anonymous. Participants completed the survey online using the mobile phone‐based interface provided by WeChat. The completion time for each questionnaire ranged from 73 to 2645 s, with an average of 276.9 ± 259.6 s. The information was automatically populated into an Excel form. Data collection ceased on July 1, 2021.

### 2.5 Participants

Participants were enrolled through the online PSES published on the official WeChat account from February 7, 2021 to July 1, 2021. The inclusion criteria were that the participants (1) had received at least one session of psychotherapy in the last month, (2) had received psychotherapy from a therapist who had a licensed qualification to practice psychotherapy issued by the government, (3) were 18−65 years old, and (4) gave informed consent. Exclusion criteria included participants being (1) in acute phase of serious mental disorders (e.g., schizophrenia, bipolar disorder, etc.) or physical illness (e.g., pneumonia, heart disease, etc.); (2) ethical faults, such as the therapist's malpractice, non‐therapeutic dual relationship, etc. and; (3) disagreements with the public release of the research data.

### 2.6 Statistical analysis

All data were integrated into one dataset. The statistical work was performed using IBM SPSS Statistics, version 22.0. Descriptive statistics were used to calculate the participants’ demographic and psychotherapy characteristics and the item scores. The internal consistency reliability was tested by Cronbach's alpha (= ɑ) coefficient and Guttman's split‐half test. The Pearson test was used to measure the correlation between the item score and the total score of PSES and between the satisfaction score and the total PSES score. Furthermore, item‐total score correlations < 0.30 were considered inappropriate for the purpose of the scale. The continuous variables among groups were compared by analysis of variance. A receiver operating characteristic (ROC) curve was generated to test the predictive validity with specificity and sensitivity. Youden's index was introduced to evaluate the ability of the scale to discriminate between clients with and without side effects. The larger the index, the stronger the authenticity of discrimination. The formula for Youden's index was as follows: (specificity + sensitivity − 1). Multi‐level factor analysis was performed using R 4.1.3 (Revelle, 2021). It was used to calculate McDonald's omega, which estimates general and total factor saturation (Zinbarg et al., 2005). ω*_t_* estimated the total reliability of the scale, whereas ω*_h_* and *ɑ* calculated the internal consistency. In this study, bilateral tests were performed, and the difference was considered statistically significant when *p* < .05.

## 3 RESULTS

### 3.1 Participants

A total sample of 456 participants tried to participate in the survey, and 420 participants ultimately met the inclusion and exclusion criteria. Table 2 shows the demographic and psychotherapy characteristics of the sample.

**TABLE 2:** Demographic and psychotherapy characteristics of the sample (*n* = 420)

Variables	Mean (SD)	Frequency
Age (in years)	35.73 (10.09)	
Gender		
Male		100 (23.8%)
Female		320 (76.2%)
Marital status		
Unmarried without partner		121 (28.8%)
Unmarried with partner		38 (9.0%)
Married		233 (55.5%)
Divorced or widowed		28 (6.7%)
Educational background		
High school or below		37 (8.8%)
University or above		258 (61.4%)
Graduate degree or above		125 (29.8%)
Occupational status		
Unemployed		53 (12.6%)
Employed		224 (53.3%)
Entrepreneur		31 (7.4%)
Other		112 (26.7%)
Psychotherapy method		
Face‐to‐face		280 (66.7)
Online		101 (24.0)
Combination of online and offline		39 (9.3)
Theoretical orientation of psychotherapy		
Psychoanalysis or psychodynamic therapy		154 (36.7)
Cognitive behavioral therapy		41 (9.8)
Family therapy		84 (20.0)
Humanistic therapy		12 (2.9)
Integrated therapy		33 (7.9)
Other therapeutic orientation		21 (5.0)
Unclear		75 (17.9)
Setting of psychotherapy		
Hospital		165 (39.3)
Social institution		150 (35.7)
Educational institution		24 (5.7)
Commonweal organization^a^		15 (3.6)
Other		66 (15.7)
Number of sessions		
1–5		122 (29.0)
6–25		176 (41.9)
26–100		99 (23.6)
>100		23 (5.5)
Number of therapists visited		
1		133 (31.7)
2		134 (31.9)
3		77 (18.3)
>3		76 (18.1)
Gender of current therapist		
Male		163 (38.8)
Female		257 (61.2)

^a^ Provides free or low‐cost psychotherapy services initiated by the government or a foundation.

### 3.2 Reliability

For all items in the scale, Cronbach's *ɑ* coefficient was .95, ω*_t_* was .97, and ω*_h_* was .8, which indicated great reliability. Only when Item 17 was deleted did Cronbach's *ɑ* coefficient slightly increase to .96 (Table 3). The Guttman split‐half coefficient was 0.88.

**TABLE 3:** Cronbach's α coefficient and correlation coefficient between items and total score (n = 420)

Items	Mean	SD	Correlation coefficient with total score	Cronbach's *α* coefficient after item was deleted	Cronbach's *α* coefficient
1. Makes my mood low	1.52	0.84	0.78	.95	.95
2. Makes my work or learning ability decline	1.33	0.74	0.80	.95	
3. Makes me dependent on psychotherapy	1.74	0.96	0.56	.95	
4. Makes me feel anxious	1.50	0.86	0.84	.95	
5. Makes me feel pessimistic or hopeless	1.31	0.75	0.83	.95	
6. Makes me want to quit psychotherapy	1.50	0.96	0.74	.95	
7. Makes me have passive emotions	1.43	0.83	0.84	.95	
8. Makes me sensitive and suspicious	1.37	0.79	0.78	.95	
9. Makes my original problem worse	1.27	0.69	0.79	.95	
10. Makes my family tense	1.22	0.62	0.69	.95	
11. Makes me feel like someone is trying to work against me	1.16	0.57	0.72	.95	
12. Makes me experience somatic discomfort	1.28	0.69	0.80	.95	
13. Makes me angry	1.54	0.93	0.75	.95	
14. Makes my relationships with others tense	1.23	0.63	0.75	.95	
15. Makes me blindly confident and optimistic	1.19	0.59	0.53	.95	
16. Makes me attack others with words or deeds	1.27	0.66	0.67	.95	
17. Makes me love the therapist	1.27	0.69	0.41	.96	
18. Makes me feel ashamed or embarrassed	1.36	0.76	0.76	.95	
19. Makes me harm myself	1.10	0.47	0.67	.95	
20. Makes my original problem remain unsolved	1.61	0.94	0.65	.95	
21. Makes my family harmful	1.11	0.43	0.64	.95	
22. Makes me hear someone talking about me, but I cannot see them	1.06	0.36	0.46	.95	
23. Makes my mood rise abnormally	1.31	0.66	0.42	.95	
24. Makes me feel inferior	1.23	0.69	0.79	.95	
25. Makes me crude or rash	1.22	0.61	0.77	.95	
Total scores	33.15	12.50	1.00		

### 3.3 Content validity

**FIGURE 1:**
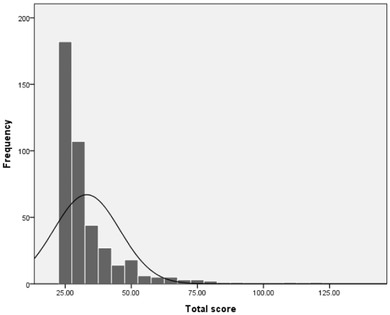
Histogram of total score frequency

The total PSES score ranged from 25 to 121, with an average of 33.15 ± 12.49 and a median of 28. The Kolmogorov–Smirnov value was 0.26, and *p* < .001, which indicates that the total score presented a normal distribution. The 95% percentile of the total score was 58.95, and the 25% percentile was 25. The histogram of the total score frequency is shown in Figure 1.

The correlation coefficient between items and the total score was between 0.41 and 0.84, in which the highest correlation coefficient was between Item 7 and the total score (*r* = 0.84), and the lowest was between Item 17 and the total score (*r* = 0.41). No correlation coefficient was < 0.30 (Table 3).

### 3.4 Concurrent validity

The correlation test between the satisfaction score and the total PSES score was used to assess concurrent validity by the Pearson test. The satisfaction score ranged from 1 to 10, with an average of 7.32 ± 1.99. The correlation coefficient between the satisfaction score and the total PSES score was −0.51 with *p* < .001, which showed a statistically significant negative correlation.

### 3.5 Discriminant validity

Of 420 subjects, 39 responded to the following question with “Yes,” and 381 responded with “No”: “Through the overall presentation of these items, do you think that psychotherapy has caused side effects for you?”. There was a significant difference in the total score of the scale between the two groups with and without side effects (*p* < .001) (Table 4).

**TABLE 4:** Comparison of the total Psychotherapy Side Effects Scale (PSES) score between the two groups with and without side effects (n = 420)

	With side effects (*n* = 39)	Without side effects (*n* = 381)	*F*	*p*
Total score	57.05 ± 20.82	30.71 ± 8.02	250.95	< .001

### 3.6 Predictive validity

**FIGURE 2:**
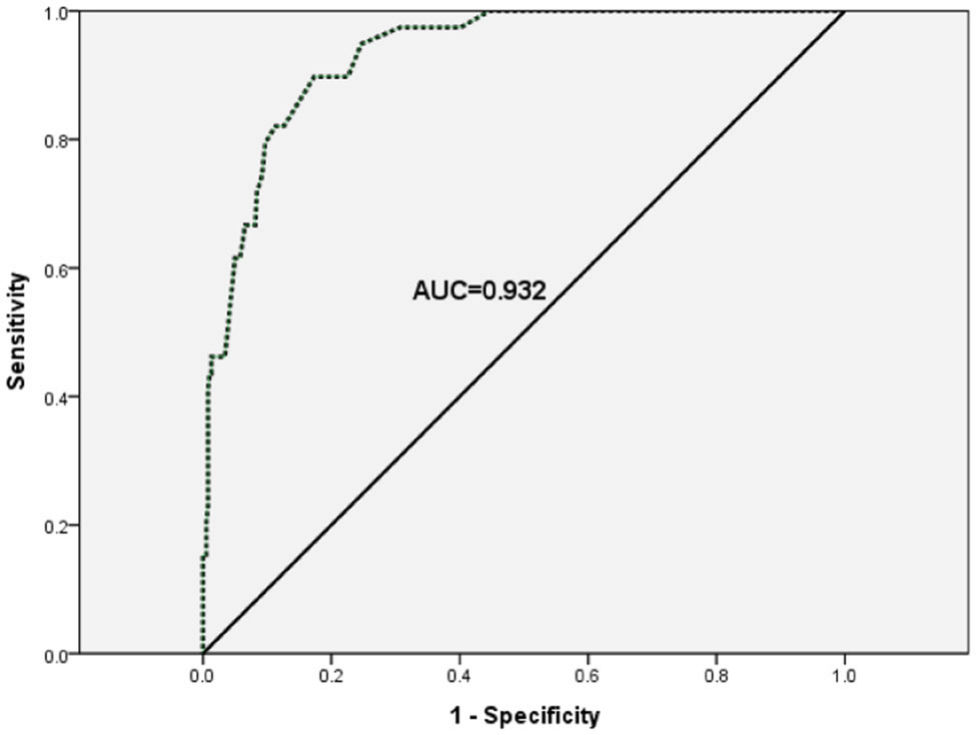
Receiver operating characteristic (ROC) curve for the prediction ability of Psychotherapy Side Effects Scale (PSES) for side effects

ROC curve analysis was performed with the total score of the PSES as the test variable and with (= 1) or without (= 0) side effects as the state variable. The results of the ROC curve demonstrated that the AUC was 0.932 with a 95% confidence interval (CI) of 0.900–0.964 (*p* < .001) (Figure 2). Next, the total score was tested by ROC curve analysis to determine which cutoff for the total score was the most conducive to distinguishing whether there were side effects. Table 5 shows that when the cutoff was 36 points, the maximum Youden's index (= 0.724) and AUC (= 0.862) could be obtained with a sensitivity of 0.897 and a specificity of 0.827. Meanwhile, the positive predictive value (PPV) was 0.347, and the negative predictive value (NPV) was 0.987. According to the proposed cutoff score, the positive rate of the PSES was 24% (101/420).

**TABLE 5:** Classification accuracy of the total score of Psychotherapy Side Effects Scale (PSES) for cases with versus without side effects (n = 420)

Cutoff	Sensitivity	Specificity	Youden's index	PPV	NPV	AUC	95% CI	*p*
33	0.949	0.753	0.702	0.282	0.993	0.851	0.800–0.902	< .001
34	0.897	0.774	0.671	0.289	0.987	0.836	0.775–0.897	< .001
35	0.897	0.811	0.708	0.327	0.987	0.854	0.794–0.914	< .001
36	0.897	0.827	0.724	0.347	0.987	0.862	0.802–0.902	< .001
37	0.872	0.843	0.715	0.362	0.985	0.857	0.793–0.921	< .001
38	0.846	0.858	0.704	0.379	0.982	0.852	0.784–0.921	< .001
39	0.821	0.874	0.695	0.400	0.979	0.847	0.775–0.920	< .001
40	0.821	0.887	0.708	0.427	0.980	0.854	0.782–0.926	< .001
41	0.795	0.903	0.653	0.456	0.977	0.849	0.773–0.926	< .001
42	0.744	0.908	0.652	0.453	0.972	0.826	0.743–0.908	< .001

Abbreviations: AUC, area under curve; CI, confidence interval; NPV, negative predictive value; PPV, positive predictive value.

## 4 DISCUSSION

The current study evaluated a new self‐report instrument for assessing the side effects of psychotherapy, the PSES. Items were generated from the themes of psychotherapy side effects discovered in previous research (Qiang et al., 2020) by our team and by using consensus among researchers and experiences of patients undergoing psychotherapy. The PSES was subsequently administered to patients receiving psychotherapy to examine its psychometric properties. The results suggested that the PSES exhibited good reliability and validity in identifying and evaluating the side effects of psychotherapy.

The SEPS has been applied to patients with obsessive‐compulsive disorder, and its Cronbach's *ɑ* coefficient was .83 (Moritz et al., 2015). In the initial attempt to use the “Psychotherapy Side Effects Questionnaire (PSEQ),” the Cronbach's *ɑ* coefficient was only .74 (Yao et al., 2020). In this study, the PSES consisted of 25 items and showed excellent internal consistency reliability with a Cronbach's *ɑ* coefficient of .95 and a Guttman split‐half coefficient of 0.880. At present, no tools for evaluating side effects of psychotherapy were tested by McDonald's omega. The values of ω*_t_* and ω*_h_* were both greater than or equal to 0.8, which indicated that the reliability of PSES was good. Compared with previous instruments, the PSES showed better reliability in the multi‐dimensional reliability test. However, the content of item 17 *“Makes me love the therapist”* might be inconsistent with the goal of the PSES. When item 17 was deleted, reliability was improved slightly. It should be no surprise that love, passions, and hate have been difficult for both clients and therapists to manage in their clinical encounters (Coen, 1996). A literature review indicated that approximately 33%−73.3% of patients reported that they felt sexually attracted to therapists; 5.2%−6.5% admitted that sexual attraction often occurred (Sonne & Jochai, 2014). Interestingly, the proportion of patients expressing sexual attraction directly to the therapist is very small (1%–2%), which is far lower than that of patients’ reports of attraction. Not surprisingly, when difficult topics perceived by patients were discussed or disclosed in therapy, treatment could continue and their alliance with the therapist could be strengthened. The reason for the low reliability of item 17 in the PSES may be the imprecision of the description, as subjects were not sure whether a feeling of love for the therapist would lead to bad results. In conclusion, we believe that the expression of the intention of item 17 should be clearer, but the item should be retained.

An effective psychotherapy side effect evaluation tool should be able to accurately distinguish the core characteristics of the target (Herzog et al., 2019). The core feature of the side effects in psychotherapy is inconsistency with the treatment goal and the infliction of burden or harm (Linden & Schermuly‐Haupt, 2014; Parry et al., 2016), which may significantly reduce patient satisfaction or the effects of psychotherapy (Whipple et al., 2003). However, there were different views concerning the side effects of psychotherapy depending on whether they were independent of the goals of therapy or whether they were unavoidable or even necessary to achieve a therapeutic effect (Linden & Schermuly‐Haupt, 2014). In any case, such side effects result in an unsatisfactory experience in psychotherapy. In the current study, there was a significant negative correlation between the results of the PSES and satisfaction, which was consistent with clinical observation and theoretical hypothesis.

To date, no measurement tools concerning psychotherapy side effects have been tested for predictive validity (Herzog et al., 2019). The most likely reason for this lack is that there are no standardized tools or gold standards for identifying side effects in psychotherapy. In our further analysis, the results indicated that the PSES could discriminate and predict side effects well based on the subjective experience of participants. The therapist is the “producer” of psychotherapy and is therefore responsible for all side effects, which may result in a perceptional bias toward positive rather than negative effects (Hatfield et al., 2010). Therefore, the actual side effects should be determined by clients rather than therapists. Meanwhile, it should be noted that the PPV of the PSES was high, while the NPV was low (0.347). Our current findings suggest that although the PSES, a self‐report scale, could measure the side effects experienced by patients in psychotherapy, it may be more suitable for the screening stage or preliminary assessment.

According to a National Audit of Psychological Therapies (NAPT) launched in England and Wales, 5.2% of patients reported long‐lasting negative effects by psychological treatment (Crawford et al., 2016). The incidence of side effects in psychotherapy was 31.1% (115/370) in a sample of Chinese patients from an online survey (Yao et al., 2020). Among the young patients who had received psychotherapy, the incidence of side effects was about 41% (Lorenz, 2021). In a recent study, the negative effects of psychotherapy were assessed by the INEP (Gerke et al., 2020). The study showed that 37.3% of inpatients and 15.2% of outpatients had at least one side effect of psychotherapy. A consensus emerged that unwanted side effects events in psychotherapy should be expected in approximately 5%–20% of patients (Linden & Schermuly‐Haupt, 2014). According to the proposed cutoff score for PSES, the incidence of side effects of psychotherapy in the study was 24%, which was close to the estimation of previous studies. However, it should be noted that many factors such as the patient's idiographic features, therapeutic orientation, the setting and the therapist could affect the incidence of side effects in psychotherapy (Crawford et al., 2016; Leitner et al., 2013; Williams et al., 2016; Yao et al., 2020). In the study, 91.2% of the participants had university education or above. The highly selective sample might generate “bottom effects” leading to the bias of the study results. In future studies, the heterogeneity of the samples will be improved to test the applicability of the PSES.

## 5 CONCLUSION

To the best of our knowledge, this was the first study to develop a new technique for assessing the side effects of psychotherapy in China. The results of this study demonstrate that the PSES shows good reliability and validity in the evaluation and identification of side effects in psychotherapy. The PSES can be used as a preliminary screening tool to facilitate the identification and management of side effects in psychotherapy by therapists and patients.

## 6 LIMITATIONS

This study constructed a fairly accurate scale for evaluating the side effects perceived by patients in psychotherapy. However, the study still faces some limitations. (1) An online survey was used to test the psychometric properties of the PSES, which may be subject to survey bias. (2) The test‐retest reliability was not tested, so it was difficult to estimate the stability of the PSES. (3) Because there was no authoritative gold standard on the side effects of psychotherapy, the accuracy of the scale needs to be further tested. (4) The structural validity of the PSES has not been studied, so the consistency between the content of the PSES and the theoretical hypothesis needs to be further improved. We will remedy the above limitations in future research and strive to develop more accurate tools.

## 7 FUTURE RESEARCH

More advanced reliability testing methods and structural validity testing for PESE need to be practiced in the future to evaluate the psychometric properties. An approved side effects scale was recommended for the evaluation of concurrent validity in the future. In addition, the reliability and validity of the PSES would be tested in the given psychotherapy to make it more suitable for clinical practice.

## AUTHOR CONTRIBUTIONS

Chen Fazhan and Zhao Xudong made substantial contributions to the conception, design, analysis, and drafting of the manuscript, ensuring that the work was appropriately investigated and resolved. Liu Liang, Feng Qiang, Ge Congcong, and Zhao Yunhan contributed to the study design and critical review of the manuscript for important intellectual content. All authors read and approved the final version of the manuscript.

## CONFLICT OF INTEREST

The authors declare no conflict of interest.

### PEER REVIEW

The peer review history for this article is available at: https://publons.com/publon/10.1002/brb3.2885.

## Data Availability

The datasets generated during and/or analyzed during the current study are available from the corresponding author on reasonable request.
